# Natural Genetic Variation in the *Caenorhabditis elegans* Response to *Pseudomonas aeruginosa*

**DOI:** 10.1534/g3.117.039057

**Published:** 2017-02-06

**Authors:** Natalia Martin, Jogender Singh, Alejandro Aballay

**Affiliations:** * Department of Molecular Genetics and Microbiology, Duke University Medical Center, Durham, North Carolina 27710; † Department of Microbiology and Molecular Genetics, Michigan State University, East Lansing, Michigan 48824

**Keywords:** wild isolate, bacterial infection, innate immunity, pathogen avoidance, *Pseudomonas aeruginosa*

## Abstract

*Caenorhabditis elegans* responds to pathogenic microorganisms by activating its innate immune system, which consists of physical barriers, behavioral responses, and microbial killing mechanisms. We examined whether natural variation plays a role in the response of *C. elegans* to *Pseudomonas aeruginosa* using two *C. elegans* strains that carry the same allele of *npr-1*, a gene that encodes a G-protein-coupled receptor related to mammalian neuropeptide Y receptors, but that differ in their genetic backgrounds. Strains carrying an allele for the NPR-1 215F isoform have been shown to exhibit lack of pathogen avoidance behavior and deficient immune response toward *P. aeruginosa* relative to the wild-type (N2) strain. We found that the wild isolate from Germany RC301, which carries the allele for NPR-1 215F, shows an enhanced resistance to *P. aeruginosa* infection when compared with strain DA650, which also carries NPR-1 215F but in an N2 background. Using a whole-genome sequencing single-nucleotide polymorphism (WGS-SNP) mapping strategy, we determined that the resistance to *P. aeruginosa* infection maps to a region on chromosome V. Furthermore, we demonstrated that the mechanism for the enhanced resistance to *P. aeruginosa* infection relies exclusively on strong *P. aeruginosa* avoidance behavior, and does not involve the main immune, stress, and lifespan extension pathways in *C. elegans*. Our findings underscore the importance of pathogen-specific behavioral immune defense in the wild, which seems to be favored over the more energy-costly mechanism of activation of physiological cellular defenses.

One of the strongest drivers of evolution is the dynamic relationship between pathogens and their hosts. While pathogens develop ever more elaborate mechanisms to establishing an infection, their hosts coevolve through the creation and diversification of an assortment of sophisticated defense mechanisms. The free living nematode *Caenorhabditis elegans* frequently encounters different pathogenic microorganisms in its natural habitat (Félix and Braendle 2010) and as a microbivore, is constantly required to counteract the effects of ingesting these potential pathogens. Recently, the first systematic analysis of the *C. elegans* microbiota was reported (Berg *et al.* 2016), elucidating the precise identity of the microbes that interact with *C. elegans* in the wild. Berg and coworkers demonstrated that the *C. elegans* native microbiome is composed mainly of Proteobacteria including, among others, the genera *Pseudomonas* (Berg *et al.* 2016).

The natural ecology, genetic tractability, simple immune system, and well-characterized nervous system of *C. elegans* make it an ideal model to gain insights into the immune pathways and behavioral mechanisms used to deal with pathogen interactions. Due to the encounters between *C. elegans* and *Pseudomonas aeruginosa* in the wild, and because of the conservation of bacterial virulence factors required to kill both *C. elegans* and mammals (Tan *et al.* 1999a; Berg *et al.* 2016), *C. elegans* is uniquely suited to elucidating the mechanisms that lead to the development of *P. aeruginosa* infection.

Under laboratory conditions, *C. elegans* responds to the pathogenic bacteria *P. aeruginosa* by utilizing a three-pronged strategy comprised of physical barriers, a behavioral response, and the activation of microbial killing pathways (Tan *et al.* 1999a; Mahajan-Miklos *et al.* 1999; Kim *et al.* 2002; Zhang *et al.* 2005; Laws *et al.* 2006; Beale *et al.* 2006; Troemel *et al.* 2006; Styer *et al.* 2008; Evans *et al.* 2008; Reddy *et al.* 2009; Chang *et al.* 2011). These last two mechanisms are not independent, but complementary, and hence can be controlled by overlapping molecular pathways. This phenomenon is observed for NPR-1, a G-protein-coupled receptor (GPCR) that is related to mammalian neuropeptide Y receptors. NPR-1 participates both in a neural circuit that controls the p38/PMK-1 MAPK pathway required for innate immunity (Styer *et al.* 2008), and in oxygen-dependent behavioral responses that affect pathogen avoidance (Cheung *et al.* 2005; Chang *et al.* 2006; Styer *et al.* 2008; Reddy *et al.* 2009).

NPR-1 is commonly found in two isoforms that differ only in a single amino acid at position 215: NPR-1 215V and NPR-1 215F (de Bono and Bargmann 1998). The NPR-1 215V isoform, which is found in the standard laboratory strain used as wild-type (N2), has increased activity relative to the 215F isoform found in most wild isolates (Coates and de Bono 2002; Rogers *et al.* 2003). These two isoforms account for different responses to food, oxygen, and other animals. Animals carrying the NPR-1 215V variant disperse evenly over the *Escherichia coli* lawn and browse for food alone (“solitary feeders”). Conversely, animals carrying the NPR-1 215F or loss-of-function alleles of *npr-1* exhibit strong hyperoxia avoidance behavior, resulting in a preference for the thickest, and hence more hypoxic part of a bacterial lawn (bordering phenotype) and aggregation into feeding groups or clumps (“social feeders”) (de Bono and Bargmann 1998).

Here, we study whether natural variation between two different isolates plays a role in host defense against the bacterial pathogen *P. aeruginosa* in *C. elegans* strain RC301, an isolate from Freiburg, Germany. We show that strain RC301 possesses enhanced resistance to *P. aeruginosa* infection compared with another *C. elegans* strain carrying the same *npr-1* allele in a wild-type background. By using a whole-genome sequencing single-nucleotide polymorphism (WGS-SNP) mapping strategy, we determined that the resistance to *P. aeruginosa* infection maps to a 5.4-Mb region between positions 7,098,350 and 12,463,435 on chromosome V. We showed that the mechanism of this resistance does not require the *C. elegans* canonical immune or lifespan extension pathways. Finally, we found that a specific pathogen avoidance behavior is solely responsible for the enhanced resistance to *P. aeruginosa* infection. Our results suggest that the implementation of pathogen-specific behavioral responses seems to be a preferred mechanism over the more energy-costly strategy of activation of physiological cellular defenses in the presence of the NPR-1 215F isoform.

## Materials and Methods

### Nematode and bacterial strains

The *C. elegans* strains are listed in Supplemental Material, Table S1 in File S2. All strains used in this study, except RC301, were backcrossed three times to the N2 strain used as wild type. The genotype of each backcrossed strain was confirmed by PCR (primer sequences provided upon request). The *C. elegans* strains were maintained at 20° on nematode growth medium (NGM) without antibiotics seeded with *E. coli* strain OP50 (Brenner 1974). The following bacterial strains were used for the experiments: *E. coli* strain HT115 (Kamath *et al.* 2003), *E. coli* strain HT115 pL4440 (Kamath *et al.* 2003), and *P. aeruginosa* strain PA14 (Tan *et al.* 1999b). All bacterial cultures were grown in Luria-Bertani (LB) broth at 37°.

### Killing assays

The *P. aeruginosa* bacterial lawns used for *C. elegans* killing assays were prepared as follows. Individual bacterial colonies were inoculated into 2 ml of LB, and grown for 8 hr on a shaker at 37°. For the partial lawn assays, 20 µl of culture was plated onto the center of a 3.5-cm plate containing modified NGM (3.5% instead of 2.5% peptone). For the full lawn assays, 50 µl of culture was spread onto the plates. All plates were incubated overnight at 37°. Twenty synchronized young gravid adult hermaphroditic nematodes were transferred to bacterial lawns, and transferred daily to a fresh lawn until progeny were no longer detected. All experiments were performed at 20° because low temperatures are known to increase the resolution of killing assays involving *P. aeruginosa*. Animals were scored at the indicated times, and considered dead upon failure to respond to touch. Animals missing from the agar plate were censored on day of loss. All experiments were performed in triplicate unless otherwise indicated. Survival was plotted using Kaplan-Meier survival curves, and analyzed by the logrank test using GraphPad Prism (GraphPad Software, Inc., San Diego, CA). A representative assay is shown in the figures. All the information of each of the assays is compiled in Table S2 in File S2.

### Lifespan assay

*E. coli* OP50 was grown for 24 hr, concentrated and heat-killed; 50 µl of this suspension was plated onto the center of NGM containing 40 µg/ml of 5-fluorodeoxyuridine (FUdR) and 50 µg/ml of ampicillin. FUdR is an inhibitor of DNA synthesis that blocks the development of progeny. The assays were performed at 20°. Animals were scored at the indicated times and considered dead upon failure to respond to touch. Animals missing from the agar plate were censored on day of loss. Three independent experiments were performed. Survival was plotted using Kaplan-Meier survival curves, and analyzed by the logrank test using GraphPad Prism (GraphPad Software, Inc., San Diego, CA).

### Lawn occupancy assays

Unless specified, lawn occupancy assays were performed as follows. *P. aeruginosa* lawns were prepared by inoculating individual bacterial colonies into 2 ml of LB, and growing them for 8 hr on a shaker at 37°. Then, 20 µl of culture was plated onto the center of a 3.5-cm plate containing modified NGM (3.5% instead of 2.5% peptone), and incubated overnight at 37°. Twenty synchronized young gravid adult hermaphroditic nematodes were transferred to bacterial lawns and counted at the indicated times for each experiment. Experiments were performed at 20°. At least three independent experiments were performed.

### RNA isolation for microarray analysis

DA650 and RC301 animals were synchronized by treating gravid adults with sodium hydroxide and bleach. Synchronized L1 animals were grown at 20° on NGM plates seeded with *E. coli* OP50. After 48 hr, the animals were rinsed off the plates with M9, washed three times with M9, concentrated and transferred to 10-cm plates containing modified NGM medium seeded with *P. aeruginosa* strain PA14. After 4 hr of incubation at 25°, the animals were rinsed off the plates, washed three times with M9 and flash-frozen in TRIzol (Life Technologies, Carlsbad, CA). Total RNA from three biological replicates was extracted using the RNeasy Plus Universal Kit (Qiagen, Netherlands). Residual genomic DNA was removed by DNase treatment (Ambion, Austin, TX). *C. elegans* Gene Expression Microarrays (Agilent Technologies, Santa Clara, CA) were used. Samples were processed according to standard Agilent protocols by the Duke Microarray Facility.

### Microarray analysis

Microarray data were analyzed using the Partek Genomics Suite (St. Louis, MO). Raw data were preprocessed, including background correction, normalization, and summarization, using robust multiarray average analysis, and expression data were log_2_-transformed. Principal component analysis (PCA) was performed to detect groupings in the data set, to identify outliers, and to evaluate whether the dataset was significantly affected by batch effects. Gene lists were created using a cutoff *P* value of <0.05, twofold change (Table S3 in File S2). For subsequent analyses, the list of upregulated genes was curated by removing duplicates and passed through the WormBase Converter (Engelmann *et al.* 2011) using the WS220 genome release as the output. For gene enrichment analysis, gene lists were selected from the literature and passed through the WormBase Converter (Engelmann *et al.* 2011) using the WS220 genome release as the output (references are listed in Table 1). The statistical significance of the overlap between two gene sets was calculated using the following on-line program: nemates.org/MA/progs/overlap_stats.html. The Representation Factor represents the number of overlapping genes divided by the expected number of overlapping genes drawn from two independent groups. A background gene list of 20,834 was used for the calculation. *P* values were calculated using the hypergeometric probability. The microarray data were deposited in the Gene Expression Omnibus database: GSE86431.

**Table 1:** Representation factors of RC301-upregulated genes

Gene Set	Genes in Set	Genes in Common	Representation Factor*^a^*	*P*-Value
CED-1 upregulated genes (Haskins *et al.* 2008)	185	4	0.6	<0.147
DAF-16 upregulated genes (Murphy *et al.* 2003)	243	9	0.9	<0.477
PMK-1 upregulated genes (Troemel *et al.* 2006)	47	2	1.1	<0.448
SKN-1 upregulated genes (Oliveira *et al.* 2009)	231	7	0.8	<0.316
SMA-6 upregulated genes (Roberts *et al.* 2010)	1469	60	1.0	<0.373

a The representation factor is the number of overlapping genes divided by the expected number of overlapping genes drawn from the group of RC301 upregulated genes, and the group corresponding to a given gene set. For details, see http://elegans.uky.edu/MA/progs/representation.stats.html.

### Mapping crosses, DNA isolation, and WGS

DA650 animals were crossed to RC301 animals, and F1 cross progeny were singled at the L4 stage. To rule out the possibility of selecting self-fertilized animals, the F1 progeny was isolated only from plates containing ∼50% males. Sixty-five F2 progeny were singled onto individual plates and allowed to self-fertilize. The F4 and successive generations were then assayed for survival and avoidance to *P. aeruginosa* as described in the sections *Killing assays* and *Lawn occupancy assays* above. Of the 65 tested strains, 13 (named S1–S13) presented an enhanced resistance to *P. aeruginosa* infection in comparison to the parental strain DA650, which was comparable to that observed for RC301, and were selected for WGS.

DA650, RC301, and S1–S13 animals were grown at 20° on NGM plates seeded with *E. coli* OP50 until starvation. Animals were rinsed off the plates with M9, washed three times, left in M9 rotating for 2 hr to eliminate food in the intestine, and washed again three times with M9. Genomic DNA extraction was performed using the Gentra Puregene Kit (Qiagen, Netherlands). DNA libraries were prepared according to the standard Illumina (San Diego, CA) protocol. The DNA from each animal was subjected to WGS on an Illumina HiSeq 2000/2500 sequencing platform using paired-end 100-nucleotide reads. Library preparation and WGS were performed at the Duke Center for Genomic and Computational Biology.

### WGS analysis

The raw WGS data were deposited in the Sequence Read Archive: BioProject ID PRJNA352286. DNA-Seq data were processed using the TrimGalore toolkit (“Babraham Bioinformatics - Trim Galore!”; http://www.bioinformatics.babraham.ac.uk/projects/trim_galore/), which employs Cutadapt (Martin 2011) to trim low quality bases and Illumina sequencing adapters from the 3′ end of the reads. Only pairs for which both reads were 20-nt or longer were kept for further analysis. Reads were mapped to the WS235 version of the *C. elegans* genome using the BWA alignment tool (Li and Durbin 2009). Duplicate read pairs were then removed from further analyses. The Genome Analysis Toolkit (GATK) (McKenna *et al.* 2010), following the best practices guide (Van der Auwera *et al.* 2002), was used to call genotypes for all samples. Variants in which the DA650 and RC301 parental strains were homozygous for alternate alleles, and where each parental strain had at least 10× coverage were used for genetic mapping. The ratio of reads that matched either the DA650 or RC301 allele were calculated for all offspring samples with a read depth of at least 10 reads. The putative effect of each called variant on the transcriptome was determined using the snpEff algorithm (Cingolani *et al.* 2012).

### RNAi-coupled survival and avoidance assays

*E. coli* HT115(DE3) bacterial strains expressing double-stranded RNA (Kamath *et al.* 2003) were grown for 8 hr in 20 ml of LB broth containing ampicillin (100 µg/ml) and tetracycline (12.5 µg/ml) at 37°. The resulting cultures were concentrated 20 times and seeded onto NGM plates containing ampicillin (100 µg/ml) and isopropyl-1-thio-β-d-galactopyranoside (2.5 mM). DsRNA-expressing bacteria were allowed to grow overnight at 37°. Gravid adult animals were placed on RNAi or vector control plates for 2 hr at 20° to synchronize their progeny. These F1 animals were grown at 20° until they reached the gravid adult stage. Gravid F1 RNAi animals were picked to a second RNAi or vector control plate and allowed to lay eggs for 2 hr at 20° to synchronize a second generation population. After reaching the young gravid adult stage, the second generation of animals was used in avoidance and/or survival assays as described in the sections Killing assays and Lawn occupancy assays above. *unc-22*(RNAi) was used in all experiments to account for the RNAi efficiency. The correct identity of all RNAi clones used in this study was verified by DNA sequencing. A total of 60 animals per condition per experiment were scored for avoidance and/or survival.

### AY125 WGS analysis

AY125 animals were grown at 20° on NGM plates seeded with *E. coli* OP50 until starvation. Animals were rinsed off the plates with M9, washed three times, left in M9 rotating for 2 hr to eliminate food in the intestine, and washed again three times with M9. Genomic DNA extraction was performed using the Gentra Puregene Kit (Qiagen, Netherlands). DNA libraries were prepared according to a standard Illumina (San Diego, CA) protocol. The DNA was subjected to WGS on an Illumina HiSeq 4000 sequencing platform using single-end 50-nucleotide reads. Library preparation and WGS were performed at the Duke Center for Genomic and Computational Biology. The raw WGS data were deposited in the Sequence Read Archive: BioProject ID PRJNA352345.

For variant calling, WGS data were subjected to strict quality control with the CutAdapt package (Martin 2011), which removed Illumina adapter sequences and low-quality base calls from the 3′ end of the reads. Only reads that were >20 nt in length after trimming were used in subsequent analyses. The reads were then aligned to a custom index containing both the *C. elegans* genome as well as the array of *uIs69* sequences (Calixto *et al.* 2010), using the BWA algorithm (Li and Durbin 2009). Duplicate reads were then removed using the Picard toolset (http://broadinstitute.github.io/picard/). The SAMtools “mpileup” algorithm (Li *et al.* 2009) was used to call variants from reads with a mapping quality of at least 30. Each variant was then annotated using the snpEff toolkit (Cingolani *et al.* 2012) to predict its functional impact according to the ENSEMBL transcriptome database (WBcel235.78) (Kersey *et al.* 2012).

### Data availability

Strains and reagents are available upon request. All sequence data have been submitted to the public repository, the Sequence Read Archive, with BioProject IDs PRJNA352286 and PRJNA352345. The microarray data have been deposited in the Gene Expression Omnibus database: GSE86431.

## Results

### RC301 animals suppress the deficiency in the NPR-1-controlled P. aeruginosa defense response by eliciting a strong pathogen avoidance behavior

**Figure 1:**
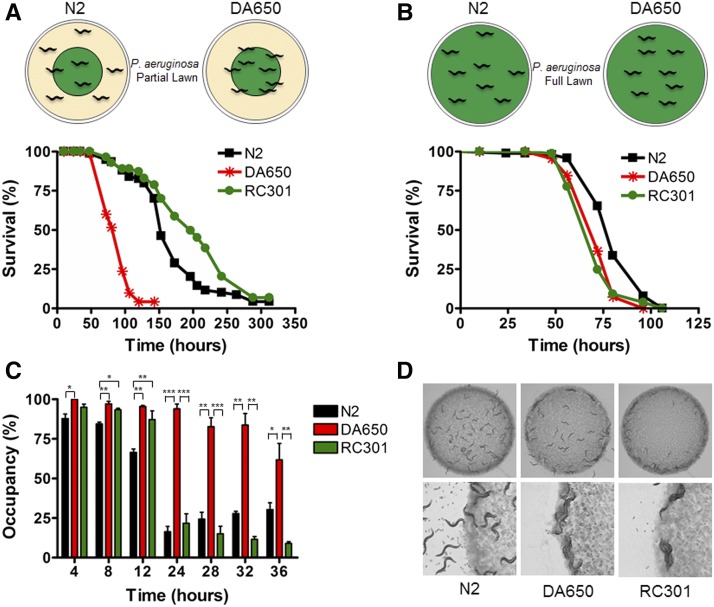
The wild isolate from Germany RC301 shows an enhanced resistance to *P. aeruginosa* infection relative to *npr-1* mutants. (A) Animals were exposed to *P. aeruginosa* under partial lawn killing assay conditions, and scored for survival. The results are a representative assay of three independent experiments; *n* = 60 animals/strain. N2 *vs.* DA650 (*P* < 0.0001), DA650 *vs.* RC301 (*P* < 0.0001), and N2 *vs.* RC301 (*P* = 0.0061) are shown. (B) Animals were exposed to *P. aeruginosa* under full lawn assay conditions and scored for survival. The results are a representative assay of three independent experiments; *n* = 60 animals/strain. WT *vs.* DA650 (*P* = 0.0002), DA650 *vs.* RC301 (NS) and N2 *vs.* RC301 (*P* < 0.0001) are shown. (C) Animals were placed on a spot of *P. aeruginosa* under standard assay conditions, and monitored over time for their presence or absence on the lawn. The graph represents the combined results of three independent experiments; error bars indicate SEM, *n* = 60 adult animals/strain. (D) Solitary and social feeding behaviors. N2, DA650, and RC301 animals were placed on a spot of *E. coli* to corroborate their feeding behavior. N2 animals were dispersed evenly across the OP50 bacterial lawn, while DA650 and RC301 aggregated together into clumps that accumulate at the border of the bacterial lawn.

The isolate from Germany (strain RC301) carries the allele for NPR-1 215F, which is responsible for altered behavioral responses to food, oxygen, and other animals, as well as reduced immune responses to bacterial infections (de Bono and Bargmann 1998; Chang *et al.* 2006; Styer *et al.* 2008). Despite carrying the allele for NPR-1 215F, RC301 animals showed an enhanced resistance to infection by the bacterial pathogen *P. aeruginosa* (Styer *et al.* 2008) (Figure 1A) compared to the DA650 strain, an RC301 derivative that carries the allele for NPR-1 215F in the N2 background (de Bono and Bargmann 1998). This observation suggested the RC301 might have evolved a mechanism to increase the defense response against *P. aeruginosa* infection in the presence of the NPR-1 215F isoform.

Initially, we performed Agilent *C. elegans* gene expression microarrays to investigate the molecular pathways require for the enhanced resistance to *P. aeruginosa* infection in RC301 animals. We aimed to identify the changes in gene expression during exposure to *P. aeruginosa*, which might promote the enhanced resistance to *P. aeruginosa* infection in the RC301 strain. To investigate this, we compared genes that were upregulated in RC301 relative to DA650 (Table S3 in File S2). Overall, this microarray data set did not reveal a significant overlap with genes previously linked to the promotion of *C. elegans* stress and innate immune responses (Murphy *et al.* 2003; Troemel *et al.* 2006; Oliveira *et al.* 2009; Roberts *et al.* 2010) (Table 1). Consistent with the lack of enrichment in genes involved in stress and lifespan extension pathways, no enhanced survival was observed between RC301 and DA650, or N2 nematodes, when fed on the nonpathogenic heat-killed *E. coli* strain OP50 (Figure S1, A and B, in File S1). Taken together, these results suggest that the enhanced resistance to *P. aeruginosa* infection observed in strain RC301 does not involve canonical *C. elegans* stress and immune pathways. Furthermore, given the role of NPR-1 in the control of the immune response via the activation of the p38/MAPK PMK-1, these results also suggest that RC301 animals elicit NPR-1-independent pathways to respond to *P. aeruginosa* infection.

*C. elegans* naturally exhibits an avoidance behavior when exposed to the bacterial pathogen *P. aeruginos*a (Zhang *et al.* 2005; Laws *et al.* 2006; Beale *et al.* 2006; Chang *et al.* 2011). Although N2 animals are initially attracted to the pathogenic *P. aeruginosa* strain PA14 (Zhang *et al.* 2005; Laws *et al.* 2006; Chang *et al.* 2011), in conditions in which they can freely enter and exit the bacterial lawn, they rapidly elicit an avoidance response to the pathogen. Consequently, the animals spend less time on the *P. aeruginosa* lawn, which partially contributes to their prolonged survival (Styer *et al.* 2008; Reddy *et al.* 2009; Shivers *et al.* 2009; Chang *et al.* 2011). In contrast to the behavior observed in N2, *npr-1* mutants have an impaired ability to avoid *P. aeruginosa*. In these animals, the lack of NPR-1-controlled responses to environmental oxygen, results in a strong hyperoxia avoidance behavior. As a consequence, *npr-1*-deficient animals exhibit a prominent preference for the border of any bacterial lawn, which is the region with the lowest oxygen levels because of the presence of more highly metabolically active bacteria (Coates and de Bono 2002). When *npr-1* mutants are exposed to *P. aeruginosa*, the hyperoxia avoidance behavior overrides the pathogen avoidance behavior. Hence, *npr-1* mutants spend most of their time in full contact with the pathogen (Styer *et al.* 2008; Reddy *et al.* 2009), and this behavioral response is partially responsible for their enhanced susceptibility to *P. aeruginosa* (Styer *et al.* 2008; Aballay 2009). In addition, *npr-1* mutants exhibit a deficient immune response to pathogens, which plays a key role in enhancing their susceptibility to infections (Styer *et al.* 2008).

To investigate whether pathogen avoidance was the mechanism responsible for the enhanced resistance of RC301 animals to *P. aeruginosa* infection, we assayed their survival on agar plates that were completely covered with *P. aeruginosa*. This condition eliminates both the lawn bordering behavior (elicited by *npr-1* mutants), and the ability to freely leave the lawn (observed in N2 animals) (Figure 1B). Animals grown on plates that were completely covered by *P. aeruginosa* died at a faster rate than those grown on plates containing a partial lawn of *P. aeruginosa* in the center of the plate (Figure 1, A *vs.* B). Whereas in the partial lawn assay (Figure 1A) RC301 animals showed an enhanced resistance to *P. aeruginosa* infection, in the full lawn conditions they displayed equivalent susceptibility compared with both DA650 and N2 animals (Figure 1B). This finding suggested that pathogen avoidance was the main mechanism responsible for the enhanced resistance to pathogen infection of RC301 animals.

To further substantiate the role of pathogen avoidance in the enhanced survival of RC301 animals, we investigated lawn occupancy over time for these three strains (Figure 1C). After 4 hr, N2 animals had already started to avoid the lawn of pathogenic bacteria, whereas DA650 and RC301 animals remained on the lawn up to 12 hr postincubation (Figure 1C). Although RC301 animals exhibited a markedly delayed avoidance in comparison to the N2 strain, at 24 hr the percentage of lawn occupancy of these two strains was indistinguishable. In contrast, the significantly higher percentage of occupancy of strain DA650 persisted up to later time points (24–36 hr). These results were unexpected, given that no difference in lawn occupancy behavior had been previously reported between strains RC301 and DA650 (Chang *et al.* 2011). We reasoned that this discrepancy could be the result of specific laboratory acquired mutations in strain DA650, or could be due to differences in the conditions used for both assays. To distinguish between these two possibilities, we repeated the lawn occupancy assay in the conditions used by Chang *et al.* (2011). As shown in Figure S2 in File S1, using these assay conditions we were able to reproduce their previously reported lawn occupancy percentages for these strains, reinforcing the general idea that small changes or variations in culture and assay conditions can dramatically affect the behavior of the animals. Given that both the survival (Figure 1A) and the lawn occupancy assays (Figure 1C) were performed under the same experimental conditions, these results demonstrate that RC301 animals enhanced their resistance to *P. aeruginosa* infection by eliciting a strong pathogen avoidance behavior toward *P. aeruginosa*.

DA650 and RC301 share the allele for NPR-1 215F, which leads to behavioral responses to food, oxygen, and other animals. Hence, both strains exhibit social food browsing (clumping), and preference for the border of the bacterial lawn (bordering) behaviors when fed the standard laboratory diet of *E. coli* OP50 (de Bono and Bargmann 1998) (Figure 1D). In fact, RC301 seems to have an even stronger bordering and clumping behavior than DA650 despite sharing the same allele for NPR-1, which could be the result of additional epistatic mutations in the RC301 background. Thus, our results indicate that the avoidance behavior exerted by RC301 animals in the presence of *P. aeruginosa* is due to a specific pathogen response that, in this case, overrides the preference of these animals for low oxygen conditions.

### Genetic mapping of the suppressor mutation(s)

**Figure 2:**
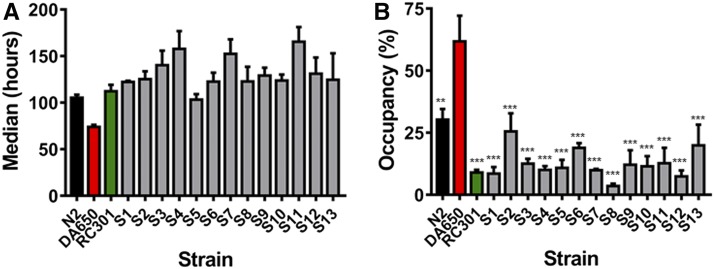
Susceptibility to pathogen and avoidance phenotypes of the strains subjected to WGS. (A) Animals were exposed to *P. aeruginosa* under standard killing assay conditions, and scored for survival. The median, which was determined for each nematode strain exposed to *P. aeruginosa*, is shown. S1–S13 are progeny of the cross of the parental strains RC301 and DA650 that showed the phenotype of interest. The graph represents the combined results of at least two independent experiments; error bars indicate SEM, *n* = 60 adult animals/strain. (B) Animals that showed an enhanced resistance to pathogen infection in comparison to the parental strain DA650 were placed on a spot of *P. aeruginosa* under the same conditions used for the survival assays, and monitored at 36 hr for their presence or absence on the lawn. The graphs represent the combined results of three independent experiments; error bars indicate SEM, *n* = 60 adult animals/strain.

To map and characterize the molecular mechanism by which RC301 animals were able to exhibit an enhanced resistance to *P. aeruginosa* relative to DA650, we used a modification of a single-step WGS-SNP mapping strategy (Doitsidou *et al.* 2010). To achieve this goal, strain RC301 was crossed with strain DA650, and the F1 progeny was isolated. Then, 65 F2 progeny were singled onto individual plates and allowed to self-fertilize. The F4 and successive generations from these 65 strains were tested in killing assays to evaluate their susceptibility to *P. aeruginosa* infection. Among 65 tested strains, 13 strains (named S1–S13) had an enhanced resistance to *P. aeruginosa* infection in comparison to the parental strain DA650 (Figure 2A, and Table S4 in File S2). These animals also displayed increased pathogen avoidance behavior (Figure 2B) that correlated with their enhanced survival.

Among the screened progeny of the crossing, 20% (13/65) presented an enhanced resistance to pathogen infection similar to that of the RC301 parental strain. This result suggested that the phenotype could be the consequence of a single recessive mutation, in which case 25% of the screened animals would be expected to have the phenotype of interest. Another possibility would be that two mutations were responsible for the suppressor phenotype of strain RC301. In this situation, if one of the mutations was recessive and the other one dominant, 18.75% of the progeny would be expected to have the phenotype of interest. The discrepancies between the observed and expected frequencies could be accounted for by the size of the population used in this study, and could potentially be overcome by increasing the number of screened animals. Because of this limitation, we could not distinguish between a single recessive mutation or two interacting suppressor mutations, one recessive and one dominant.

**Figure 3:**
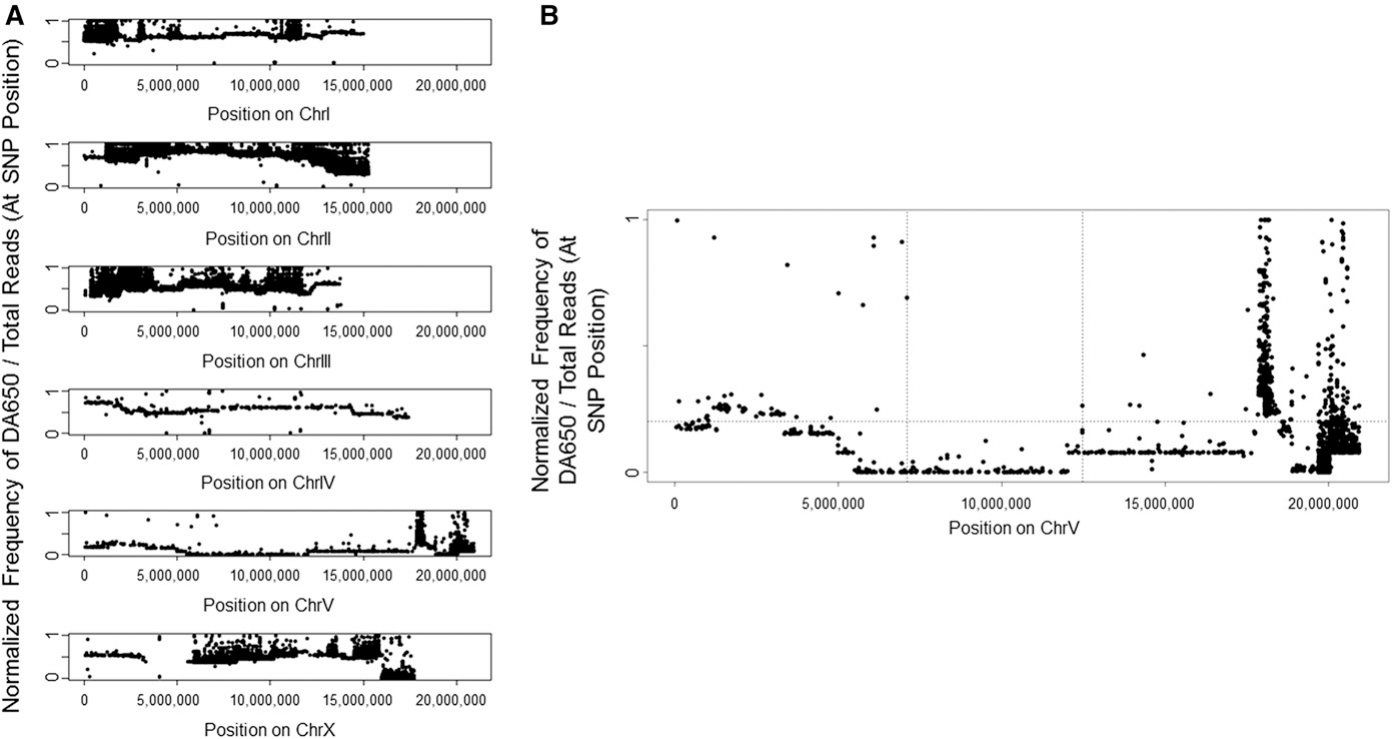
WGS-SNP data for all chromosomes. (A) The plot shows the normalized ratio of the DA650 allele/total reads at each mapped SNP position in the genome. The mapped SNP positions are those distinguishing the parental strains DA650 and RC301. (B) The plot shows the enlarged mapping region of chromosome V. The mapping region was defined by the physical location of the two consecutive mapping SNPs located furthest apart from one another using a frequency = 0.2 as filtering criteria (dotted horizontal line). Dotted vertical lines located at positions 7,098,350 and 12,463,435 delimit the mapping region.

The 13 animals (S1–S13) that exhibited the phenotype of interest were selected for WGS. Due to meiotic recombination, it is expected that in regions that are unlinked to the mutation(s), the parental chromosomes will recombine in an unbiased manner. However, the closer a SNP locus is to the mutation(s), the more infrequent a recombination event will be between that SNP and the mutation. Hence, DA650 variants will be underrepresented in regions that are closer to the selected mutation(s), whereas RC301 SNPs will cosegregate very close to the mutation(s). We took advantage of the SNPs distinguishing the parental strains DA650 and RC301 to perform the WGS data analysis. The representation of the normalized frequency of DA650/total reads (at each SNP position) (Figure 3A) revealed an even distribution of SNPs at frequencies of ≥0.5 for chromosomes I–IV, but no regions enriched in RC301 SNPs. For chromosome X, we mostly found an even distribution of SNPs at frequencies ≥0.5. However, between position 4,045,190 and 5,864,922, there is a region in which no SNPs distinguished the two parental strains. This region includes the *npr-1* gene, and accounts for the fact that strain DA650 is an RC301 derivative that was generated by successive outcrosses to the N2 strain (de Bono and Bargmann 1998).

We observed low frequencies of DA650 SNPs across the central region of chromosome V. This enrichment in RC301 SNPs suggests that this chromosome harbors the phenotype-causing mutation(s). Based on previous studies (Doitsidou *et al.* 2010; Minevich *et al.* 2012), a threshold of 0.2 was established to define the mapping region. Accordingly, the mapping interval was defined between position 7,098,350 and 12,463,435 (5.4 Mb) on chromosome V (Figure 3B).

### Candidate gene analysis

Within the 5.4 Mb defined as the mapping region, analysis of the variants observed among the 13 sequenced strains revealed no premature stop codons or frameshifts mutations. However, several mutations that did not affect protein coding regions were found. Additionally, we were able to detect 14 variants that caused missense mutations in protein-coding regions. A list of the affected genes with the corresponding protein changes is shown in Table 2.

**Table 2:** Polymorphisms in the region between positions 7098350 and 12463435 of chromosome V

Position	N2 Allele	RC301 Allele	Gene Name	Protein Change
7,278,103	G	A	*C13A2.5*	E424K
7,374,927	A	C	*C03G6.2* (*srx-56*)	K124T
7,725,357	T	A	*ZK105.8*	F166Y
7,929,847	C	T	*Y97E10B.14*	H39Y
8,983,180	T	C	*F59E11.14* (*str-86*)	S141P
9,053,715	A	C	*C25E10.8*	T36P
9,528,381	T	C	*C50F4.1*	I225T
10,280,570	C	A	*Y22F5A.5* (*lys-2*)	T213K
10,352,457	C	A	*K07C5.4*	H92Q
10,468,570	C	T	*F22E12.4* (*egl-9*)	P16S
10,784,870	A	G	*D1054.8*	N145S
11,143,547	T	C	*C06H2.7*	S236P
11,820,883	A	G	*D2023.2* (*pyc-1*)	K444E
11,840,650	T	C	*R13H4.1* (*nphp-4*)	I655T

We began by addressing the possibility of a single recessive mutation, in which case two scenarios are feasible: (i) a recessive loss-of-function mutation, or (ii) a recessive gain-of-function mutation. Although possible, recessive gain-of-function mutations are rare (see, for example, Kernan *et al.* 1991; García-Añoveros *et al.* 1995). Hence, to identify the gene/s responsible for the phenotype of interest, we initially performed a systemic RNA interference (RNAi) screen to knockdown these 14 candidate genes. To achieve this goal, the parental strain DA650 was fed with vector control RNAi, or RNAi for each of the potential candidate genes (Table 2), and assayed to evaluate its avoidance phenotype.

**Figure 4:**
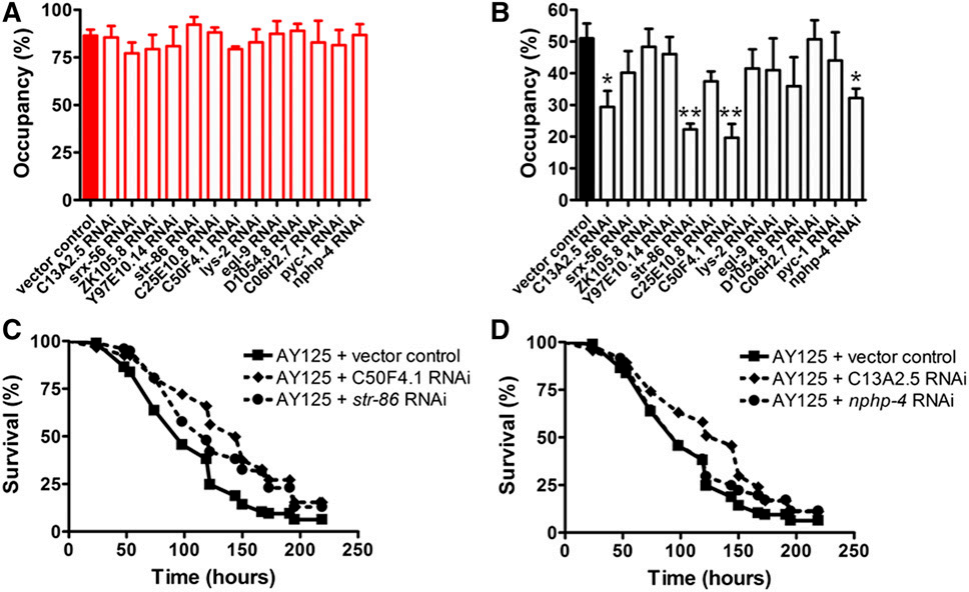
Systemic and neuron-specific RNAi screening of loss-of-function phenotypes of the 13 candidate genes. DA650 (A) and AY125 (B) animals were fed vector control RNAi or RNAi for each of the potential candidate genes for two generations, and then placed on a spot of *P. aeruginosa* under standard assay conditions, and monitored at 24 hr for their presence or absence on the lawn. The graphs represent the combined results of three independent experiments; error bars indicate SEM, *n* = 60 adult animals/strain. *P < 0.05 and **P < 0.001 was determined using the Student’s *t*-test based on the results of at least three independent experiments. (C, D) AY125 animals were fed vector control RNAi or RNAi for each of the potential candidate genes for two generations, exposed to *P. aeruginosa* under standard killing assay conditions, and scored for survival. The graphs represent the combined results of three independent experiments. Differences were statistically significant for all cases but *nphp-4* RNAi. Vector control *vs. str-86* (*P* = 0.0001), vector control *vs. C50F4.1* (*P* < 0.0001), and vector control *vs. C13A2.5* (*P* = 0.0004) are shown. *n* = 60 adult animals/strain.

Knockdown of gene *K07C5.4* resulted in a developmental delay. Such developmental defect was not observed in strain RC301, and thus it is unlikely that the phenotype-causing mutation affected this gene. Although we cannot rule out the possibility of an epistatic allele in strain RC301 that suppresses the developmental delay of variation in *K07C5.4*, this gene was excluded from further analysis. As shown in Figure 4A, none of the RNAi treatments caused a significant difference in the avoidance phenotype when compared with the vector control condition.

Although *C. elegans* exhibits systemic RNAi (Tabara *et al.* 1998; Timmons and Fire 1998), it has a delayed and deficient response to RNAi in neurons (Kamath *et al.* 2001). Due to the possibility that the phenotype-causing mutation affects a neuronal gene, the previous result is not sufficient to rule out these candidate genes. To address this possibility, we generated strain AY125 [*npr-1(g320)*; *sid-1(pk3321)*; *uIs69* V], a DA650 derivative generated by cross to strain TU3401 (Calixto *et al.* 2010). This strain is deficient for SID-1, the transmembrane protein required for systemic RNAi, while overexpressing *sid-1* from the pan-neuronal *unc-119* promoter. Using strain AY125, we performed a neuron-specific RNAi screen of the 13 candidates (Figure 4B). We found that strain AY125 exhibited enhanced avoidance to *P. aeruginosa* compared with strain DA650 (Figure 4A, red bar *vs.* Figure 4B, black bar). This enhanced avoidance to *P. aeruginosa* of strain AY125 decreased the overall sensitivity of the avoidance assay under this neuron-specific RNAi condition. Among the 13 candidate genes, four exhibited increased avoidance to *P. aeruginosa* compared with the control: *C13A2.5*, *str-86*, *C50F4.1*, and *nphp-4*. Given that the enhanced resistance to *P. aeruginosa* infection of strain RC301 correlated with the increased avoidance to the pathogen, we further investigated the effect of the knockdown in a neuron-specific manner for these four candidate genes using pathogen survival assays. The two candidates that caused the most significant difference in avoidance behavior, *str-86* and *C50F4.1*, also significantly enhanced resistance to *P. aeruginosa* infection (Figure 4C). Consistent with the avoidance results (Figure 4B), neuron-specific knockdown of *C13A2.1* also increased resistance to *P. aeruginosa* infection compared with control conditions (Figure 4D). However, *nphp-4* RNAi had minimal to no effect on strain AY125 when compared with control conditions (Figure 4D). Because the enhanced resistance to *P. aeruginosa* infection is a consequence of the strong pathogen avoidance behavior exhibited by RC301 animals, these results suggest that, while *C13A2.5*, *str-86*, and *C50F4.1* could potentially be the candidate genes affected by the phenotype-causing mutation, *nphp-4* is an unlikely candidate.

Although the level of false positives in RNAi experiments has been reported to be extremely low (<1%) (Kamath *et al.* 2003), the possibility of false positives remains feasible. To investigate this possibility, we used clustered regularly interspaced short palindromic repeat (CRISPR)/CRISPR-associated protein-9 nuclease (Cas9) genome editing to generate mutations in the two candidate genes that caused the most significant differences in the avoidance and survival to *P. aeruginosa*: *str-86* and *C50F4.1*. Strains carrying two different null alleles of each of these genes (Figure S3, A and B, and Figure S4, A and B, in File S1) were then crossed to DA650 animals to generate the corresponding double mutants, strains AY126 to AY129 (Table S1 in File S2). Next, we assessed the avoidance and survival of these double mutant strains in response to the pathogen *P. aeruginosa*. Surprisingly, neither the mutation in *str-86* nor the mutation in *C50F4.1*, was able to rescue the lack of the pathogen avoidance phenotype caused by the NPR-1 215F isoform (Figure S3C and Figure S4C, in File S1).

Knockdown of *C13A2.5*, in a neuron-specific manner, also increased avoidance of strain AY125 compared with the control conditions. Although this effect was not as significant as that observed for *str-86* and *C50F4.1*, the possibility of *C13A2.5* being the phenotype-causing mutation remained feasible. To address this possibility, we used a null allele of *C13A2.5* to construct strain AY130, an *npr-1(g320)*; *C13A2.5 (gk792531)* double mutant. Strikingly, as observed for the other two candidate genes, the mutation in *C13A2.5* did not rescue the lack of the avoidance phenotype caused by the *npr-1(g320)* allele (Figure S5 in File S1). Thus, the increased pathogen avoidance and resistance, resulting from the knockdown of these three genes in a neuron-specific manner, could have been the consequence of RNAi off-target effects. As mentioned previously, the estimated rate of false-positive RNAi phenotypes is extremely low (<1%) (Kamath *et al.* 2003). However, this percentage is based on experiments performed using *C. elegans* N2 strain. Thus, we cannot rule out the possibility that the use of mutants or overexpressing strains, such as TU3401, alter the false positive rate. Alternatively, these results could be a consequence of the decreased sensitivity of the *P. aeruginosa* avoidance assay with strain AY125. As mentioned above, AY125 is a derivative of strain DA650 generated by a cross with strain TU3401 (Calixto *et al.* 2010). The extrachromosomal array in strain TU3401 was integrated by gamma radiation in a chromosomal location to the right of *dpy-11* on chromosome V (Howe *et al.* 2016), which indicates that it is adjacent to the right end or within our mapping region. Consequently, the presence of the array could be altering the expression or mutating gene/s within the mapping region in strain AY125. Alternatively, mutations caused by the gamma radiation procedure could have remained linked to the array even after multiple crosses performed to homogenize the background. These scenarios could explain the lack of a correlation between the suppression phenotypes observed for certain candidate genes in the neuronal RNAi screen (Figure 4B), and the phenotypes of the respective mutants (Figure S3C, Figure S4C, and Figure S5 in File S1).

To further investigate the possibility of the array interrupting a gene or regulatory sequence within the mapping region, and/or the presence of additional mutations linked to the array, we sequenced strain AY125 and analyzed the WGS data to detect SNPs, insertion and deletions (INDELs), and the genomic position of the integration of the array. The SNPs generated by comparison of strain AY125 to the N2 reference genome are listed in Table S5 in File S2. As shown, we did not detect SNPs in strain AY125 that affected any of the candidate genes (Table 2 and Table S5 in File S2). However, gamma radiation causes a high frequency of large multigene deletions and complex genomic rearrangements (*e.g.*, translocations, crossover suppressions, among others) (Epstein and Shakes 1995). Given the nature of the mutations caused by gamma radiation, and the difficulty related to variant callers to accurately detect INDELs [for a discussion, see Hu (2014)], it is possible that certain mutations could have been overlooked during the analysis. Despite our efforts, we did not succeed in accurately identifying the genomic position in which the array is integrated.

Although we could not detect additional mutations affecting any of the candidate genes in strain AY125, the 20% of the animals presenting the phenotype of interest during screening, the lack of suppression of the *npr-1* phenotype for all tested candidate genes and the low recombination rate across most of chromosome V, strongly suggest that the variation in pathogen avoidance behavior between DA650 and RC301 strains could be a complex trait involving more than one locus in the same chromosome.

## Discussion

Our findings demonstrate that variation between two *C. elegans* isolates, RC301 and DA650, plays a role in the response of *C. elegans* to the bacterial pathogen *P. aeruginosa*. We found that strain RC301 displayed enhanced resistance to *P. aeruginosa* infection compared with DA650. We took advantage of the variation between these two strains to study the phenotypic and genetic determinants of this pathogen resistance. The two *C. elegans* strains, RC301 and DA650, encode the same isoform of the GPCR related to the mammalian neuropeptide Y receptors NPR-1 215F, while the remaining of their genomes are divergent. Most wild isolates of *C. elegans* harbor the NPR-1 215F isoform; the variation 215V found in the standard laboratory strain used as N2 seems to be the result of the adaptation to the laboratory environment (Weber *et al.* 2010). NPR-1 variants have been associated with a plethora of phenotypes, including aggregation, bordering, speed of locomotion, burrowing, ethanol tolerance, response to ambient oxygen, and behavioral and immune responses to several bacterial pathogens, among others (de Bono and Bargmann 1998; Davies *et al.* 2004; Gray *et al.* 2004; Cheung *et al.* 2005; Rogers *et al.* 2006; Styer *et al.* 2008; Reddy *et al.* 2009; Andersen *et al.* 2014; Nakad *et al.* 2016). Some of these traits, such as the deficiency in immune and behavioral responses, are certainly detrimental for the survival of *C. elegans* in the diverse microbe-rich habitats in which it is primarily found (Félix and Braendle 2010). As demonstrated by recent microbiota studies, worms in such environments are continually interacting with microbial pathogens (Dirksen *et al.* 2016; Berg *et al.* 2016), which usually infect them via oral uptake during feeding. Thus, it is anticipated that wild isolates could have evolved mechanisms to overcome the presence of the immune and avoidance phenotypes caused by the NPR-1 215F isoform. The present comparison of the wild isolate from Germany, RC301, with strain DA650 is suitable to unveil additional traits that could be important for the survival of *C. elegans* in nature.

As a bacterivore in the wild, *C. elegans* is constantly facing the “to eat or not to eat” dilemma. Once *C. elegans* comes in contact with bacterial pathogens, two different scenarios are possible: (i) activation of physiological and cellular defenses to combat the infection, or (ii) avoidance of the infectious threat by escaping from pathogen-rich environments. While the recognition of microbial cues is the first and common step in both strategies, the subsequent mechanisms differ. *C. elegans* can activate several dedicated pathways that regulate immunity, oxidative, and xenobiotic stress responses and increase longevity (recently reviewed in Rodriguez *et al.* 2013; Kim 2013; Cohen and Troemel 2015). While extremely effective, this strategy is also highly costly in terms of energy (Schmid-Hempel 2003), and can cause self-damage in the absence of precise control (Singh and Aballay 2009). Alternatively, *C. elegans* can mount a behavioral immune defense to avoid contact with the infectious organism by moving away from the pathogen-rich area (Schulenburg and Müller 2004; Pradel *et al.* 2007; Styer *et al.* 2008; Reddy *et al.* 2009). This strategy is advantageous because it saves energy while minimizing the infection. Interestingly, we found that the activation of the main immune, stress, and lifespan extension pathways of *C. elegans* is not required to promote the enhanced resistance to *P. aeruginosa* infection in the wild isolate, strain RC301. In contrast, the energy-efficient strategy of a pathogen-specific avoidance behavior is exclusively responsible for this phenotype. The NPR-1-mediated avoidance of *P. aeruginosa* has been linked to the oxygen-dependent behavioral response (Styer *et al.* 2008; Reddy *et al.* 2009). However, given that both strains used in the present study share the NPR-1 215F isoform, we can rule out an effect of the NPR-1-controlled oxygen-dependent responses in the pathogen avoidance phenotype of strain RC301. Although further studies including other wild isolates are required, our results highlight the importance of these pathogen avoidance behaviors in the wild suggesting that in natural niches they could be the preferred mechanism to overcome the immune and avoidance phenotypes caused by the allele for NPR-1 215F. Our findings also emphasize the importance of the use of different genetic backgrounds to address relevant aspects of *C. elegans* biology.

The screen performed during the one-step WGS mapping strategy used to map the causative mutation yielded 20% (13/65) of the animals with enhanced resistance to pathogen infection similar to the parental strain RC301. Several possibilities could explain the observed percentages: (i) a single recessive mutation could be responsible for the observed phenotype, in which case 25% of the progeny would be expected to behave like the parental RC301 strain; (ii) the phenotype could be due to the presence of recessive plus a dominant mutation, resulting in an expected 18.75% of the progeny with the parental RC301 phenotype; or (iii) the causative mutation could be semidominant; however, this alternative would imply a very low penetrance of the allele. The differences between the observed and expected frequencies could be due to the size of the population used for this analysis. Thus, we could not distinguish between the alternatives (i) and (ii). This limitation could potentially be solved by increasing the population size selected for the screening. Although (iii) is feasible, the penetrance would have to be significantly low for the causative mutation to be dominant, making this option highly unlikely. In such a case, the only difference between a single recessive and a semidominant mutation would be the phenotype of the heterozygote animals; in the first situation they would have a phenotype similar to the DA650 parental strain, and in the second, an intermediate phenotype would be expected between the two parental strains. Although we observed intermediate phenotypes during screening, these were most likely due to the intrinsic variability of the assay.

The use of this WGS-SNP mapping approach (Doitsidou *et al.* 2010; Minevich *et al.* 2012) allowed us to determine that the resistance to *P. aeruginosa* infection in strain RC301 maps to a 5.4-Mb region between positions 7,098,350 and 12,463,435 on chromosome V. However, a substantially larger region on chromosome V exhibited low recombination frequencies, making it more difficult to define the mapping interval. This larger area of chromosome V with lower recombination frequencies could be due to the population size used for this study, or to the enhanced resistance of RC301 to *P. aeruginosa* infection resulting from a potentially complex trait of two or more loci on chromosome V.

Within the 5.4-Mb mapping interval, 14 genes harbored a SNP between the RC301 and DA650 strains. To determine if the loss of function of any of these 14 genes was responsible for the enhanced resistance to *P. aeruginosa* infection phenotype, we knocked them down systemically by RNAi. No difference was observed between the RNAi treatments compared with the control condition, indicating that the loss of function of these genes is not responsible for the enhanced resistance to pathogen infection, at least systemically. However, knockdown in a neuron-specific manner revealed three candidates (*str-86*, *C50F4.1*, and *C13A2.5*) that could suppress the avoidance and survival phenotypes. Given the neuronal expression of NPR-1 and considering that the nervous system can respond in milliseconds to a wide range of environmental cues, it is not surprising that the compensatory mechanism leading to the pathogen avoidance response and to the enhanced resistance to pathogen infection phenotype of RC301, would be neuronally encoded. In fact, we constructed transcriptional reporters for *str-86* and *C50F4.1*, and observed their expression in the nervous system. Despite the suppression phenotypes observed following the neuron-specific knockdown of these three genes, the use of nonsense mutations in those genes did not correlate with the observed phenotypes. There are two possible explanations for this discrepancy: (i) the phenotypes observed following neuron-specific knockdown could be the consequence of RNAi off-target effects; and (ii) the strain used for the neuron-specific knockdown, AY125, could carry mutations that interact with the phenotypic effects of the knockdown of these genes. We evaluated the RNAi specificity using the web-service E-RNAi (Horn and Boutros 2010) and were unable to identify other target sequences for these RNAi. Additionally, the extremely low false-positive rates estimated for RNAi phenotypes (<1%) (Kamath *et al.* 2003) make this alternative unlikely. As described previously, strain AY125 is a derivative of strain DA650, which was generated by a cross with strain TU3401 (Calixto *et al.* 2010). It is possible that either the site of integration of the *P_unc-119_sid-1* extrachromosomal array, or mutations generated by the gamma rays used to integrate the array in strain TU3401, could have epistatic effects with the knocked down genes. Despite sequencing strain AY125, we could not determine with confidence the exact chromosomal location of the integrated array. Thus, we cannot eliminate the possibility that the presence of the array could either affect the expression or the sequence of gene/s within the mapping region in strain AY125. Another possibility is that gamma radiation-induced mutations remained linked to the array even after extensive backcrossing this strain. Analysis of the WGS data for strain AY125 did not reveal SNPs or INDELs in any of the candidate genes. The latter mutations, which are most likely to arise after gamma radiation (Epstein and Shakes 1995), are still challenging to reliably detect by current callers due to difficulties related to distinguishing genomic regions with no sequencing coverage from those that are true deletions (Sarin *et al.* 2010; Hu 2014). Given the limitations of this approach, other strains (such as *eri-1*; *lin-15b* or *nre-1*; *lin-15b* double mutants) (Sieburth *et al.* 2005; Schmitz *et al.* 2007) would have to be used to test the neuronal knockdown of these genes to indisputably eliminate a potential effect of the AY125 background in the observed phenotypes.

Although possible, recessive gain-of-function mutations are extremely rare (see, for example, Kernan *et al.* 1991; García-Añoveros *et al.* 1995), and thus this option is unlikely. However, further experiments are required to conclusively exclude this alternative, or the possibility that the causative mutation is in a noncoding region.

In summary, our findings demonstrate that the activation of a pathogen-specific avoidance behavior seems to be the preferred mechanism to compensate for the presence of the NPR-1 215F isoform in the wild isolate RC301, and that the activation of key immune, stress and longevity pathways is not required. Although we were not able to pinpoint the genetic determinant/s of the phenotype-causative mutation(s), we narrowed it down to a 5.4-Mb interval on chromosome V. Additional experiments are needed to identify the gene/s responsible for the compensatory mechanism in the German wild isolate RC301.

## Supplementary Material

Supplemental material is available online at www.g3journal.org/lookup/suppl/doi:10.1534/g3.117.039057/-/DC1.
