# Supplementary material for: Natural Genetic Variation in the Caenorhabditis elegans Response to Pseudomonas aeruginosa
Source: G3 (Bethesda). 2017 Feb 6;7(4):1137–47. doi: 10.1534/g3.117.039057 (PMC5386862; doi:10.1534/g3.117.039057)
Supplement: Supplementary file 1 [file 1137FileS1.pdf]

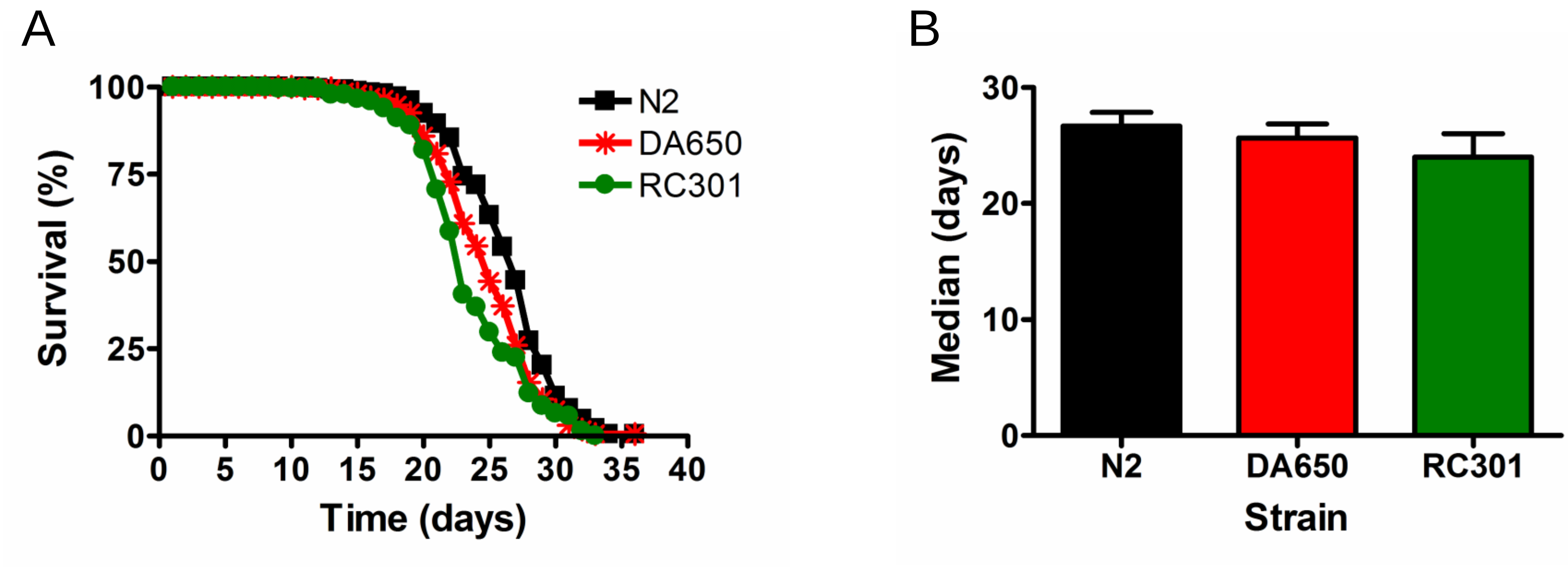

Figure S1. (A) Animals were exposed to heat-killed *E. coli* and score for lifespan. The survival graphs represent the combined results of three independent experiments; n = 350 animals/strain. N2 versus DA650 ($P < 0.0001$), DA650 versus RC301 ($P = 0.0493$) and WT versus RC301 ($P < 0.0001$) are shown. **(B)** The median, which was determined for each nematode strain exposed to heat-killed *E. coli*, is shown. The graph is representative of the three independent experiments shown in panel A, error bars indicate s.e.m.

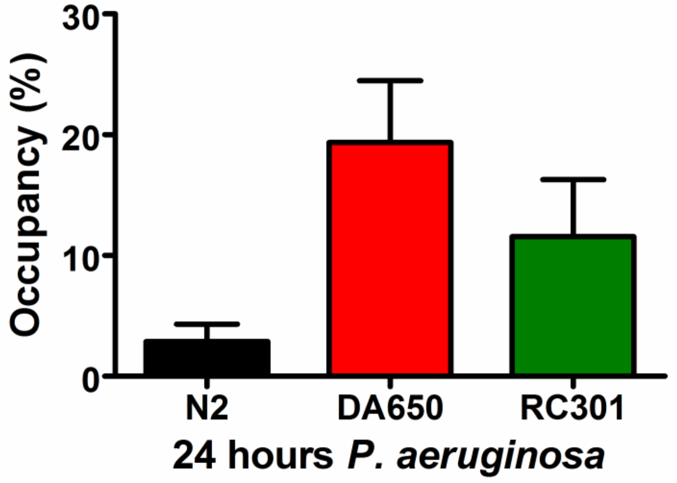

Figure S2. N2, DA650, and RC301 animals were placed on a spot of *P. aeruginosa* under the same conditions used by Chang and coworkers (Chang et al., 2011) and monitored at 24 hours for their presence or absence on the lawn. The graphs represent the combined results of three independent experiments, error bars indicate s.e.m. n = 60 adult animals/strain.

A

```
                 446                            475
str-86 (WT)      AATGGAGTATTTCAACGGAGGGTATTTAAT
str-86 (ec320)   AATGGAGTATTT-AACGGAGGGTATTTAAT
                              △
                         gggtatttaatgttt

                 439                            478
str-86 (WT)      TTAAAAAAATGG(16bp)GTATTTAATGTT
str-86 (ec321)   TTAAAAAAATGG------GTATTTAATGTT
```

B

```
WT              MLTFTIRCEQLGVVVALFSNTMLFYLLTFKANTSYGAYRRLMFSYTIVELMYSVISVMSGMMAH
STR-86(ec320)   MLTFTIRCEQLGVVVALFSNTMLFYLLTFKANTSYGAYRRLMFSYTIVELMYSVISVMSGMMAH
STR-86(ec321)   MLTFTIRCEQLGVVVALFSNTMLFYLLTFKANTSYGAYRRLMFSYTIVELMYSVISVMSGMMAH
WT              STETSFVVFDLYEGYVSRYLAPIFLIDCAFYFTLILLLVVHFIYRYVVVCDFKKMEYFNGGYL
STR-86(ec320)   STETSFVVFDLYEGYVSRYLAPIFLIDCAFYFTLILLLVVHFIYRYVVVCDFKKMEYLTGI*-
STR-86(ec321)   STETSFVVFDLYEGYVSRYLAPIFLIDCAFYFTLILLLVVHFIYRYVVVCDFKKMGI*-----
WT              MFWVFGSAACGVSMCILKFFAFPETERRSIELSEEFSLYYNLTMDQVVYNGPNYYICNENDECE
STR-86(ec320)   ----------------------------------------------------------------
STR-86(ec321)   ----------------------------------------------------------------
WT              MPLKDWTAMMILSNGLIFSIVIMLYCGYRCCVKLNKNDKQTSCRTVDLQKQLMIALIIQSVIPI
STR-86(ec320)   ----------------------------------------------------------------
STR-86(ec321)   ----------------------------------------------------------------
WT              IFMYIPILLLFITPMFKIGLGPYVNIAMATLSIYPPIDQFAIIYVIKDFRIGVKDFFKCGKKSD
STR-86(ec320)   ----------------------------------------------------------------
STR-86(ec321)   ----------------------------------------------------------------
WT              NTSSSTIFTTRLSLTKSHLSL*
STR-86(ec320)   ----------------------
STR-86(ec321)   ----------------------
```

B

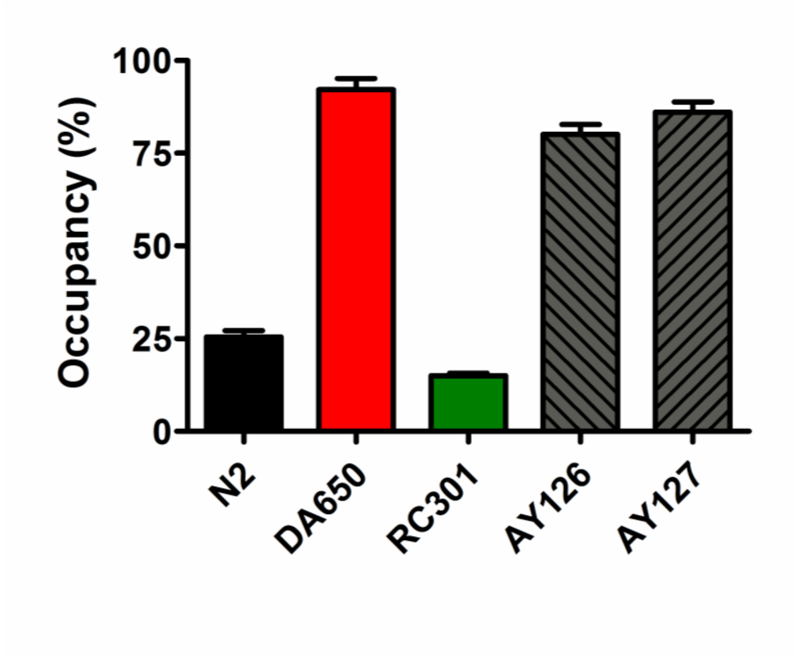

Figure S3. (A-B) Schematic of the CRISPR/Cas9 generated *str-86* mutant alleles and STR-86 variants. **(C)** N2, DA650, RC301, AY126 [*str-86(ec320); npr-1(ad650)*] and AY127 [*str-86(ec321); npr-1(ad650)*] animals were placed on a spot of *P. aeruginosa* under standard assay conditions and monitored at 24 hours for their presence or absence on the lawn. The graph represent the combined results of three independent experiments, error bars indicate s.e.m. n = 60 adult animals/strain.

A

```
                    13                                175
C50F4.1 (WT)        GCATTAATTGCA(139bp)AGGGCATTGCAA
C50F4.1 (knu123)    GCATTAATTGCA------aAGGGCATTGCAA

                    73                             102
C50F4.1 (WT)        ATCAATGGTGTCACAACCGTGGAGTTAGAG
C50F4.1 (knu124)    ATCAATGGTGTCACA-CCGTGGAGTTAGAG
                                   △
               tgtccgatgtccgtgtccgtgtccgtggaccgtggtgt
```

B

```
WT                 MFITALIAKELGLSRVKELNPTVTINGVTTVELETAEGLFRVHLVRDDQDVIVKLEEGSKLAAY
C50F4.1(knu123)    MFITALIAKGIAKISKTFNSKFKCTTDIKYYKIIVTPITVSFFNGSNFKRILQIF*--------
C50F4.1(knu124)    MFITALIAKELGLSRVKELNPTVTINGVTVRCPCPCPWTVVYRGVRDSGGPLPSAFGER*----
WT                 YNSLFWHEISTFEKIPIPSAHFNRSFVVTEDENKHSVYVTQFLKKGSVRTKLGLPELEQVASQI
C50F4.1(knu123)    ----------------------------------------------------------------
C50F4.1(knu124)    ----------------------------------------------------------------
WT                 AKLHAVNSKTINKQFSLNVQENYGNIISFKKKIQKELIEVLETAVTTTEVADYFMNPSSVIEKV
C50F4.1(knu123)    ----------------------------------------------------------------
C50F4.1(knu124)    ----------------------------------------------------------------
WT                 GILTHYLEDWKDEDADEKEKHNVIAHGRLTAEICRFDEDGNLVEITEWENIHLGNPVEDLANLI
C50F4.1(knu123)    ----------------------------------------------------------------
C50F4.1(knu124)    ----------------------------------------------------------------
WT                 VSSADVDIRRKKFMKIFQVYFYALVDYYPPKYQLHDLKRWFQEYQPTVLINGIESLLFTLSEGS
C50F4.1(knu123)    ----------------------------------------------------------------
C50F4.1(knu124)    ----------------------------------------------------------------
WT                 DDVKQDAARRWETALNDTVDFLTGNYISDNEHPFLSQKENDD*
C50F4.1(knu123)    -------------------------------------------
C50F4.1(knu124)    -------------------------------------------
```

B

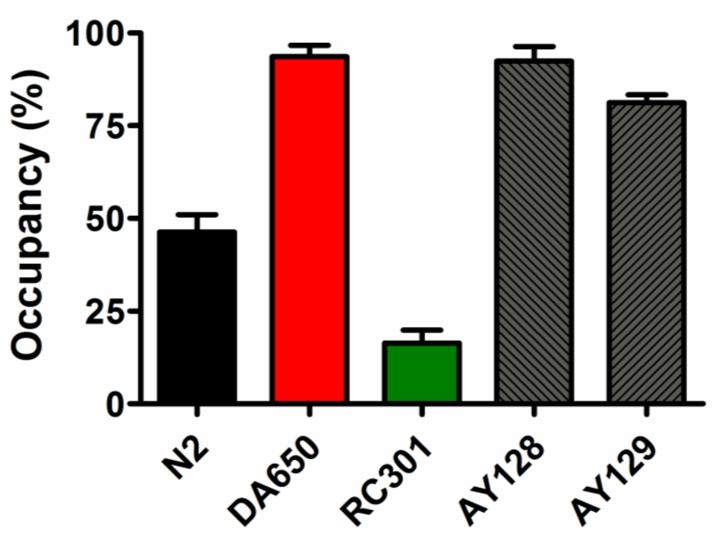

Figure S4. (A-B) Schematic of the CRISPR/Cas9 generated *C50F4.5* mutant alleles and C50F4.1 variants. **(C)** N2, DA650, RC301, AY128 [*C50F4.1(knu123);npr-1(ad650)*] and AY129 [*C50F4.1(knu124);npr-1(ad650)*] animals were placed on a spot of *P. aeruginosa* under standard assay conditions and monitored at 24 hours for their presence or absence on the lawn. The graph represents the combined results of three independent experiments, error bars indicate s.e.m. n = 60 adult

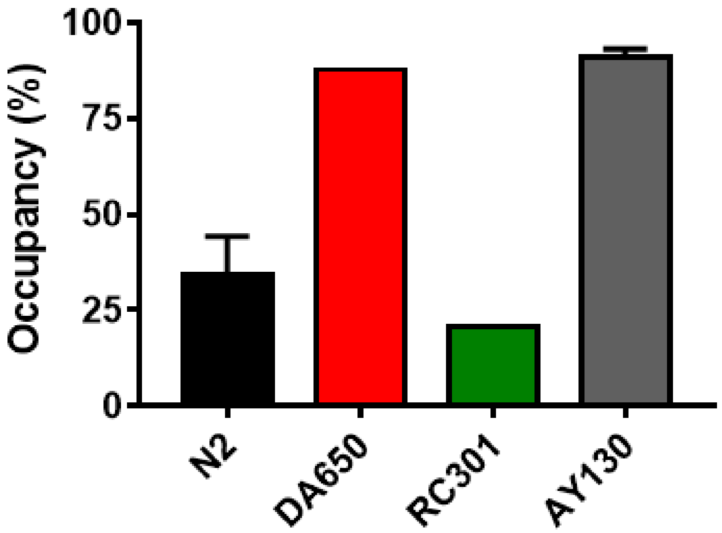

Figure S5. N2, DA650, RC301 and AY130 [*C13A2.5(gk792531); npr-1(ad650)*] animals were placed on a spot of *P. aeruginosa* under standard assay conditions and monitored at 24 hours for their presence or absence on the lawn. The graph represents the combined results of three independent experiments, error bars indicate s.e.m. n = 60 adult animals/strain.
